# Age and area predict patterns of species richness in pumice rafts contingent on oceanic climatic zone encountered

**DOI:** 10.1002/ece3.3980

**Published:** 2018-04-24

**Authors:** Eleanor Velasquez, Scott E. Bryan, Merrick Ekins, Alex G. Cook, Lucy Hurrey, Jennifer Firn

**Affiliations:** ^1^ School of Earth Environmental and Biological Sciences Faculty of Science and Engineering Queensland University of Technology Brisbane Qld Australia; ^2^ Queensland Museum Brisbane Qld Australia; ^3^ School of Agriculture and Food Sciences The University of Queensland Brisbane Qld Australia

**Keywords:** community assembly, general dynamic model of oceanic island biogeography, long‐distance dispersal, propagule pressure, species–area curve

## Abstract

The theory of island biogeography predicts that area and age explain species richness patterns (or alpha diversity) in insular habitats. Using a unique natural phenomenon, pumice rafting, we measured the influence of area, age, and oceanic climate on patterns of species richness. Pumice rafts are formed simultaneously when submarine volcanoes erupt, the pumice clasts breakup irregularly, forming irregularly shaped pumice stones which while floating through the ocean are colonized by marine biota. We analyze two eruption events and more than 5,000 pumice clasts collected from 29 sites and three climatic zones. Overall, the older and larger pumice clasts held more species. Pumice clasts arriving in tropical and subtropical climates showed this same trend, where in temperate locations species richness (alpha diversity) increased with area but decreased with age. Beta diversity analysis of the communities forming on pumice clasts that arrived in different climatic zones showed that tropical and subtropical clasts transported similar communities, while species composition on temperate clasts differed significantly from both tropical and subtropical arrivals. Using these thousands of insular habitats, we find strong evidence that area and age but also climatic conditions predict the fundamental dynamics of species richness colonizing pumice clasts.

## 1 INTRODUCTION

The globe is experiencing its sixth mass extinction event, and considerable evidence suggests that native biodiversity is being lost as a result of human activities (see Ceballos et al., 2015). Concurrent with this loss is the increasing homogenization of biotas across countries and even continents via the widespread transport and establishment of species (Cardinale et al., 2012; Hooper et al., 2012). Due to increasing numbers of non‐native and potentially invasive species arriving in new habitat, understanding the fundamental processes that regulate differences in diversity levels has arguably never been as important. A fundamental theory first proposed in 1963 to explain patterns in diversity, the theory of island biogeography (TIB), has been tested numerous times as a framework for predicting the dynamic processes acting on insular populations (MacArthur & Wilson, 1963, 1967). Key elements of this theory, for example, the species–area relationship (SAR), have become instrumental in the field of conservation for managing fragmented landscapes (Diamond, 1975; Diamond et al., 1976; Margules, Higgs, & Rafe, 1982; Whittaker et al., 2005).

Use of the SAR as a starting point and predictive tool for ecological research is frequently undertaken to see whether the expected relationship of increasing area results in an increased number of species being able to reside within that area (MacArthur & Wilson, 1963; Simberloff & Abele, 1976). This expected relationship between species richness and habitat area as predicted by the SAR has been hypothesized to not only occur because of increased resources but also because the number of habitats (or habitat heterogeneity) increases, while at the same time a larger population has the flow on effect of reduced extinction rates (Brown & Kodric‐Brown, 1977; Dengler, 2009; Goldstein, Carson, & Eriksen, 2014; MacArthur & Wilson, 1963; Simberloff, 1976; Triantis et al., 2008). However, the SAR has remained somewhat equivocal in relation to its underlying processes and application (Gilbert, 1980; Lomolino, 2000; Lynch & Johnson, 1974; Triantis et al., 2008). This is due to two key difficulties with measuring the SAR: (1) habitat size can be challenging to estimate in natural ecosystems; and (2) studies have tended to focus on single‐specific taxa (e.g., bats (see Mendenhall, Karp, Meyer, Hadly, & Daily, 2014) or birds (see Diamond, 1969)) as a surrogate for all biodiversity (Gilbert, 1980).

In concert with principles detailed in the TIB, other theories have developed simultaneously including the incorporation of multiple measures of biodiversity such as gamma, beta, and alpha diversity as detailed by Whittaker (1960). Whittaker (1960) determined the total species diversity in the landscape (gamma diversity) is comprised of: (1) alpha diversity: being the mean species richness which exists within certain sites or habitats within the landscape, and (2) beta diversity: being the differences in species richness or diversity between the different sites or habitats. Together, the TIB and the concepts of alpha and beta diversity can be used to understand the similarities and differences in diversity when comparing insular habitats and the ecological drivers which influence the observed resultant biotic composition (MacArthur & Wilson, 1963; Whittaker, 1960). The TIB predicts that richness of biodiversity (or alpha diversity) in isolated environs or habitats at the local scale are explained by species turnover as a function of area and through processes of immigration, speciation, and extinction that will eventually reach a dynamic equilibrium of species, because resources and space become saturated (Keppel, Buckley, & Possingham, 2010; MacArthur & Wilson, 1967; Simberloff & Wilson, 1969; Whittaker, 1960), while beta diversity allows us to compare the differences between species composition between similar and different habitat types in a broader landscape or oceanic context (Anderson, Ellingsen, & McArdle, 2006; Whittaker, 1960).

Since the inception of the TIB, aspects of this theory have been tested numerous times and the original ideas have evolved. For example, Whittaker, Triantis, and Ladle (2008) proposed the general dynamic model of oceanic island biogeography (GDM) to include processes operating on both geological and evolutionary timescales (Borregaard et al., 2016; Whittaker et al., 2008). The GDM expands on TIBs original concepts of immigration and extinction by including processes of oceanic island lifecycle (originally proposed by Darwin (1859)) and the impact these processes have on island biota both in time and space (Darwin, 1859; Whittaker et al., 2008). The GDM proposes that species richness increases quickly (upon island formation) before reaching saturation (as niche space becomes limited), because of interactions between age (time) and area, while isolation removes certain species from the pool of immigrants (Whittaker et al., 2008).

**Figure 1:**
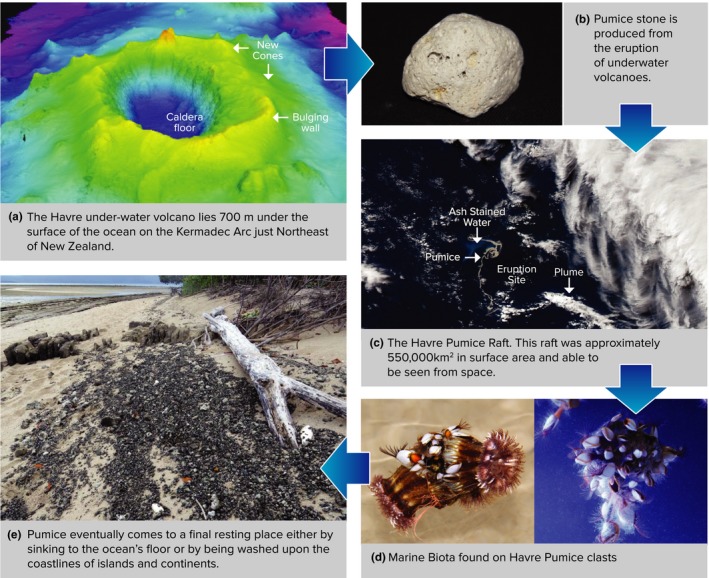
Conceptual diagram of pumice ontogeny. This conceptual diagram depicts the journey of a pumice clast from (a) the Havre under‐water volcano which erupted in July 2012, forming approximately four trillion pieces of floating pumice stone. (b) This pumice then formed what is known as a pumice raft (c) which was so large it could be seen from space. The raft slowly dispersed over the open ocean driven by winds and currents, and while this occurred, marine biota (d) colonized the empty surface of the clasts. Finally (e), pumice is either washed onto the coastlines of islands and continents or sinks due to waterlogging or biofouling

Pumice rafts present a unique model system to understand how patterns of biodiversity change over time in insular habitats because habitat size and biodiversity can be measured; and pumice rafts from individual events are formed from the same submarine explosion (Bryan et al., 2012; Jutzeler et al., 2014). These rafts are floating masses of individual pumice stones and can range in size dependent upon the force of the eruption from a few square kilometers to thousands of square kilometers floating on the surface of the ocean (Bryan, 1971; Bryan et al., 2004, 2012). Pumice rafts provide the ideal opportunity to test the TIB, in particular the SAR (with the inclusion of additional biotic and abiotic drivers) as each pumice stone acts as a “mini‐island”; whose size (measured using the surface area of individual clasts), age (measured as the length of time (in days) from eruption until stranding on coastlines), and the trajectory path it has taken influence its exposure to different oceanic climatic zones (referred to from now on as climatic zone), which can all be measured as pumice clast ontogeny (see Figure 1) (MacArthur & Wilson, 1963, 1967; Whittaker et al., 2008).

To test fundamental concepts surrounding the SAR, we investigated three key questions: (1) Do area and age predict epibiont richness or alpha diversity which forms on pumice clasts and does one have a stronger influence? (2) How does the influence of area and age change the resultant biodiversity or alpha diversity forming on pumice clasts that were collected from different climatic zones? and (3) Does pumice rafted community assembly (beta diversity) change for pumice that originated from different eruptions or that took different trajectories? Based on the SAR, we predict that larger pumice stones will carry more species because of increased habitat heterogeneity, lower probabilities of extinction, and increased clast stability in the water column (Bravo et al., 2011; Hart & Marshall, 2012; MacArthur & Wilson, 1963; Whittaker et al., 2008). Larger pumice clasts likely facilitate increased rates of immigration because of higher resource availability or just by chance because of a higher “target effect” (Lomolino, 1990; MacArthur & Wilson, 1967). Target effect increases the chance of immigration due to size—larger islands or habitats are simply more likely to be “found” by potential colonizers than smaller habitats (Whittaker et al., 2014). Taking elements from the ideas contained in the GDM (i.e., age), we also predict that older pumice clasts are likely to carry more species (Whittaker et al., 2008). As habitat space is small on individual pumice clasts and hence can become saturated at low numbers of species and the amount of time pumice spends in the ocean is relatively short, for example, when compared to geological or evolutionary time scales (Anderson, 1999; Whittaker et al., 2008). We also predict that the trajectory path of the pumice rafts influences strongly the abiotic conditions (e.g., sea surface temperature, island or shallow reef encounters) experienced by the raft and the species pool the raft is exposed to, particularly when differing climatic zones (e.g., temperate, subtropical, and tropical) are traversed, and will thus influence species richness patterns found (Gray, 2002; Thiel, Guerra‐García, Lancellotti, & Vásquez, 2003; Wichmann, Hinojosa, & Thiel, 2012). The influence of climatic zones is therefore expected to alter the influence of area and age dependent on climatic zone traversed. Further to this, the multivariate dispersion (or beta diversity) of pumice rafted communities is also expected to vary dependent on climatic zone encountered. As beta diversity is a measure of community similarity or dissimilarity among sampling units that are grouped based on the point of collection (climatic zone), we would expect that clasts of similar area and age from the same climatic zone would be comprised of similar communities (Anderson et al., 2006).

## 2 MATERIAL AND METHODS

### 2.1 Home reef raft and trajectory

Home Reef (referred to from hereon as “Home”), Tonga, erupted from 7 to 16 August 2006 after 22 years of dormancy producing a floating mass of pumice containing approximately 2.5 × 10^12^ pumice clasts (for more detail of raft trajectory see Bryan et al., 2012). As concluded by Bryan (et al. 2012), a conservative estimate of one‐third of the pumice raft produced is expected to have reached the Eastern Australian coastline (Bryan et al., 2012).

### 2.2 Havre volcano raft and trajectory

The Havre submarine volcano, located adjacent to the Kermadec Islands north‐east of New Zealand, erupted on July 17, 2012 (Jutzeler et al., 2014; Priestley, 2012; Schiel, Kingsford, & Choat, 1986; Wunderman, 2012). The resulting pumice raft containing approximately 3–4 × 10^12^ pumice clasts began arriving to the eastern Australian coastline after approximately 8 months (for more details of the raft and its spread see Priestley, 2012; Wunderman, 2012; Jutzeler et al., 2014). After Bryan et al. (2012), we assume that approximately 1/3 of the Havre pumice raft arrived to the eastern Australian coastline being 1.16 × 10^12^ pumice clasts.

All pumice clasts arriving to the eastern Australian coastline had been colonized, thus, taking a conservative 1:1 relationship of clast to organism ratio, more than one trillion individuals have been transported via these pumice rafts (Bryan et al., 2012).

### 2.3 Pumice characteristics

Pumice is known to contain many small inclusions which trap air, leading to buoyancy and longevity at the surface of the ocean (Bravo et al., 2011; Thiel & Haye, 2006). The inclusions cause pumice to have a heterogeneous surface, filled with many vesicles and crevices which increase surface area of individual clasts and further aids colonization by marine biota (Bravo et al., 2011). (For more information on pumice clast formation and stability please see Appendix S1.)

### 2.4 Sampling design

Samples of pumice stone were opportunistically collected from strandlines on beaches and coastlines in various locations (Bryan et al., 2012). Collection of pumice attempted to capture a diverse range of sizes and biodiversity as determined by the collector and what was available in the opportunistic stranding of the pumice clasts (Bryan et al., 2012). (For details of pumice raft collection sites and dates please see Table S1.)

Pumice clasts from the Home and Havre eruptions originated in the tropical and subtropical climatic zones, respectively (Bryan et al., 2012; Cole, 2001; Schiel et al., 1986). Pumice clasts collected on coastlines were considered as belonging to one of three climatic zones: subtropical, tropical, and temperate based on the Australian Coastal Biogeographic and Climatic Zone classification system (Bucher & Saenger, 1994). As it was not possible to determine the exact trajectory of the pumice clasts and all of the different climatic zones they may have traversed, we have used their collection point to determine the most influential climatic zone on final species assemblages. We acknowledge that while this approach does not account for all possible shallow marine ecosystem encounters or sea surface temperature effects, it still provides a good approximation of the oceanic climatic zone pumice spent a majority of its time afloat in.

For each clast, we estimated total habitat area using digital calliper measurements of maximum length and width and calculated the available surface area (or available habitat) using the surface area of a rectangular prism (formula: 2(wl + hl + hw) (where w = width, l = length, h = height)), or sphere (formula: 4πr^2^ (where r = radius)). We tested for strength of correlation between sphere and prism measurements and found correlation values to be greater than 95%. We chose then to use sphere in all further analyses (for details of pumice clast sizes, please see Figure S2).

We identified all biota to their lowest level of classification, and where further formal classification could not occur, evident differences between individuals of the same family were undertaken to allow division into functional types, referred to as epibionts (Bryan et al., 2012; Martins, Faria, Furtado, & Neto, 2014; Wahl, 1989). For example, worms of family Serpulidae were subdivided into functional types of white, pink, and gray‐colored calciferous tubes. The term “epibiont richness” is used in place of species richness for this study as the pumice rafted organisms were not always able to be identified down to species level (Bryan et al., 2012). The presence or absence of all biota was recorded for each pumice clast.

### 2.5 Data analyses

We developed linear mixed‐effects models (MEMs) using R (version 3.1.2; Foundation for Statistical Computing) and the lme4 library (Bates, Mächler, Bolker, & Walker, 2015) to investigate the relationship between the response variable of epibiont richness and the fixed effects of: area and age; with a random effect structure of: event, place of collection, and date collected for the climatic zones tropical and subtropical. For samples collected in the temperate zone, the random‐effects structure consisted of place of collection and date collected because pumice was not collected from temperate waters for the Home event. Due to the different scales for the fixed effects age and area, these were centered prior to modeling using the R (3.1.2) “center” function (Cade, 2015). Once a model was fit, residual plots were inspected for model fit and if the residual plots were reasonable, then it was concluded that the model provided a satisfactory fit to the data. Model comparisons were undertaken with the MuMIN package (Barton, 2013). Models were evaluated with the corrected Akaike information criterion (AIC_c_) using the model averaging function (Johnson & Omland, 2004), and we considered models within four AIC_c_ units to be competing models (Burnham & Anderson, 2002). The AIC_c_ evaluation of component models is undertaken in this case due to the unbalanced design of the dataset resulting from this natural experiment (Burnham & Anderson, 2002). Parameter estimates using a random intercept structure from the simplest candidate model, following the principles of parsimony, were then plotted to compare effect sizes using the package coefplot2 (Bolker, 2012). We chose a random intercept structure of date collected nested within location code and then within event as multiple collections occurred at the same location on different dates across both events. We then generated *R* ^2^_c_ and *R* ^2^_m_ values for each model using the “lmer” package for each candidate model in order to ascertain how much of the variation was explained by the models both including and excluding the random effect structure (Bates et al., 2015).

Prior to analysis, data from the eruptions of Home and Havre were combined. This was done as a majority of pumice from the Home eruption were from the subtropical climatic zone and no clasts from the temperate climatic zone were collected. We ran the MEMs relating to climatic zone of collection by separating the data into each climatic zone, and this was done as using climatic zone as a fixed effect in our analysis resulted in oversaturation of the model.

We then performed tests of the differences and similarities between epibiont community composition, in relation to area, age, climatic zone, and location. Using the Primer 7 software package (version 7.0.10, with add‐on: PERMANOVA+ 1) utilizing the permutational multivariate analysis of variance (PERMANOVA), pair‐wise test and PERMDISP functions (see Table S2, S3 and S4) (Anderson, 2001; Anderson et al., 2006; Clarke & Gorley, 2015; McArdle & Anderson, 2001). For the PERMDISP analysis, only data from the Havre event were analyzed as there were too few collection points from different climatic zones for the Home event, with a majority from subtropical and only a few from tropical to perform this test (Anderson et al., 2006). Nonmetric multidimensional scaling (NMDS) was used to visualize these differences in assembly (Clarke & Gorley, 2015). Tests were conducted to compare one continuous quantitative covariable: area; and four factors: event (Home vs. Havre), age (early, middle, late), climatic zone (tropical, subtropical, and temperate), and location (nested within event, age, and climatic zone) of different clasts to determine whether epibiont communities differed based on these parameters (see Table S2). Age of the pumice clasts was grouped into early (pumice that arrived in the first 3 months based on first arrivals to the coastlines of continents and islands), middle (pumice that arrived after 3 months but before 11 months), and late arrivals (pumice which arrived after 11 months). The area of pumice clasts was extremely right skewed and hence was transformed to log (base 10) creating a normal distribution (see Figure S2). Quantiles of 0.25, 0.5, and 0.75 were calculated for the distribution of sphere sizes resulting in four size‐class groups: a1, a2, a3, and a4 (from smallest to largest) forming a factor we called “area.q” to allow for ease of graphical representation. Averages for each combination of location × area.q were calculated for the biotic data, and an average of location × area.q for the log (base 10) sphere values was also calculated. This process resulted in effectively treating each combination of location and the associated distribution of size classes of pumice that arrived at this location as a replicate for our study.

Comparisons between averaged log (base 10) sphere as a quantitative covariate, event, age, and climatic zone was conducted using epibiont biota presence/absence data which was averaged by area and location (area.q) and a Bray–Curtis resemblance matrix. This was performed using unrestricted permutation of raw data and number of permutations set to 9999. Initial comparisons yielded several inestimable terms, due to unbalanced properties of the design. These inestimable terms resulted from combinations in the model matrices that yielded cells that simply did not exist due to imbalance in the cell structure in the design, and hence, these terms were removed before further analysis was conducted.

## 3 RESULTS

We recorded more than 116 epibiont groups from 10 phyla after surveying 5,279 pumice clasts from the Havre and Home eruptions collected at 29 locations and within three climatic zones (temperate, subtropical, tropical) (see Table S5).

### 3.1 Do area and age predict epibiont richness (alpha diversity) and does one have a stronger influence?

**Figure 2:**
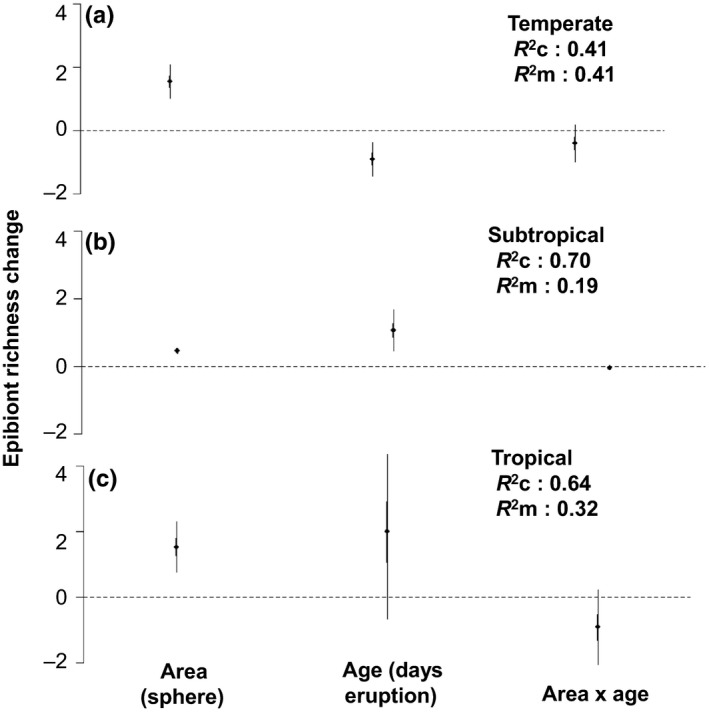
Mixed‐effects model regression estimates for the combined data of Home and Havre (*n* = 5,279) events within climatic zones: a) temperate (*n* = 70), b) subtropical (*n* = 5,043), and c) tropical (*n* = 166); showing epibiont richness as a function of the covariates of: age (days since eruption), area (an estimation of available habitat calculated for each individual pumice clast using the surface area of a sphere), and a combined effect of age x area. The error bars displayed in the above figure are the standard error as derived from the coefficient estimates in the model. The *R* ^2^_m_ values indicate the amount of variation explained by the model without the random effects, while the *R* ^2^_c_ values indicate the amount of variation explained by the model with the random effects

Model comparisons using combined data for Home and Havre showed that age is the most influential covariate for epibiont richness overall, with model weights between 0.37 and 0.91 in models calculated separately for each climatic zone; area was shown to be the second most influential predictor overall (model weights 0.09–0.32), with the interaction of area × age being the least influential (model weights 0.01–0.30) (see Table 1). It should also be noted that the AIC_c_ values for models produced for area or age are within four AIC_c_ points for subtropical and tropical climatic zones and as such are considered to be equivalent models. While for temperate these models did not fall within four AIC_c_ points, they were not considered equivalent. Using the *R* ^2^ value to assess the component models, we found that a high degree of model variation was explained with our component models which tended to increase with the inclusion of random effects except for the temperate climatic zone (see Figure 2).

**Table 1:** Results of model comparison using Akaike information criterion (AIC_c_) values to identify factors explaining variations in epibiont richness between the pumice clasts within climatic zones using the surface area of a sphere as an estimate for available habitat

Climatic zone	Effect	*df*	logLik	AICc	Delta	Weight
Temperate (*n* = 70)	Age	8	−153.48	325.32	0.0	0.91
	Area (sphere)	6	−158.34	330.02	4.7	0.09
	Area × age	N.A. because the models that explained the most variation were clearly age followed by area.				
Subtropical (*n* = 5,043)	Age	8	−9,312.48	18,640.98	0.00	0.84
	Area (sphere)	7	−9,315.22	18,644.46	3.47	0.15
	Area × age	6	−9,319.24	18,650.50	9.51	0.01
Tropical (*n* = 166)	Age	8	−414.58	846.08	0.00	0.37
	Area (sphere)	7	−415.83	846.36	0.28	0.32
	Area × age	6	−416.97	846.48	0.39	0.30

N.A., not available.

Climatic zones include temperate (*n* = 70), subtropical (*n* = 5,043), and tropical (*n* = 166) of area, age, and area × age.

### 3.2 How does the influence of area and age change for pumice clasts that were collected from different climatic zones?

We found epibiont richness varied depending on: age, climatic zone, and area. For both tropical and subtropical climatic zones, epibiont richness increased with both time and size of the pumice clast (see Figure 2). In the temperate zone, the relationship between epibiont richness and time was negative suggesting epibiont richness may be lost over time as pumice drifted into cooler climates, but area had a positive correlation with epibiont richness.

**Figure 3:**
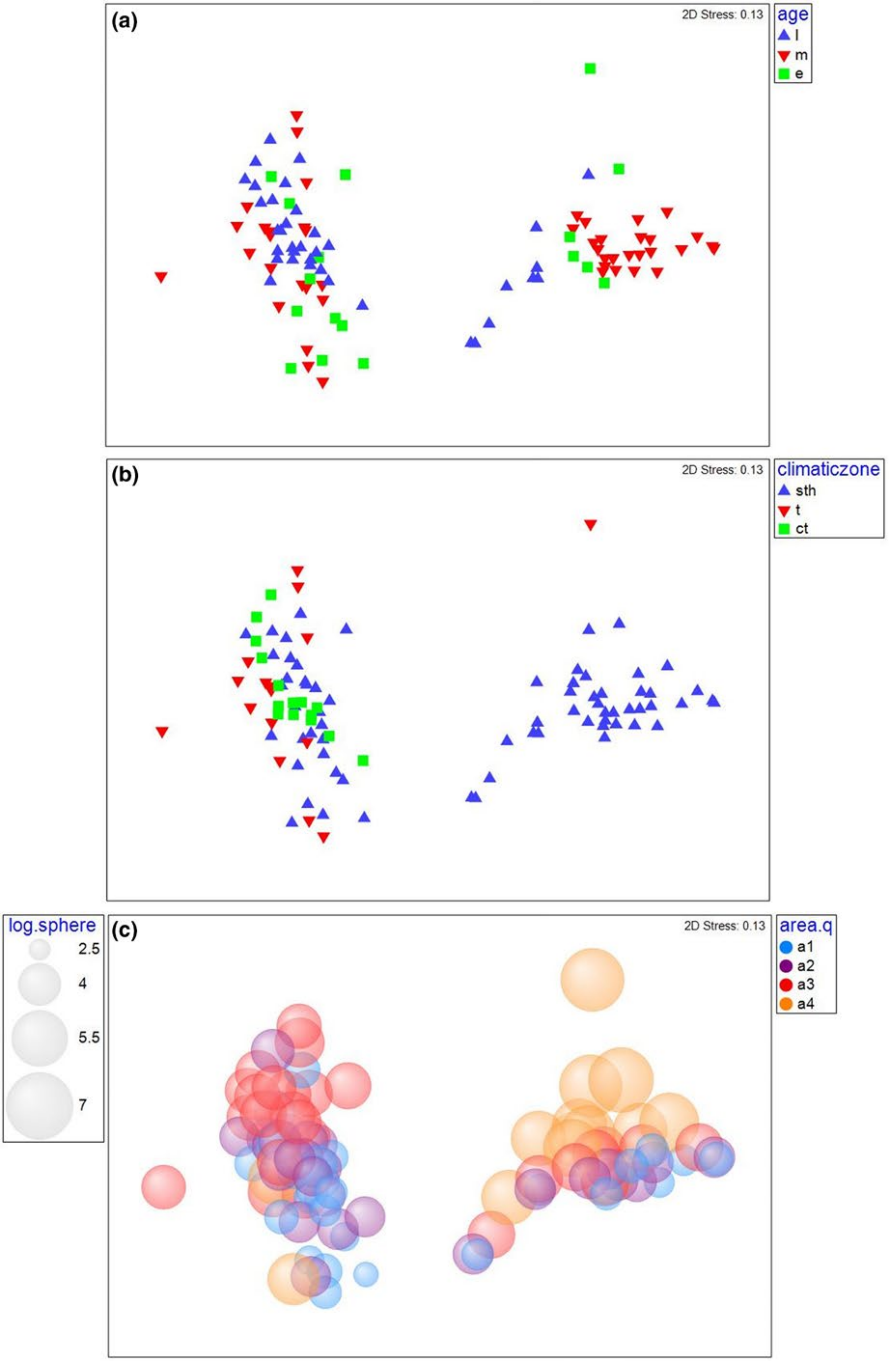
NMDS of a) age (where e = early, m = middle, and l = late arrivals of pumice clasts to beaches on the east coast of Australia and Pacific Islands), b) climatic zone (where sth = subtropical, t = tropical, and ct = temperate stranding locations), and c) area effect on pumice rafted community composition distinguished by location for two events Home (right‐hand cluster) and Havre (left‐hand cluster). Note for panel b), the effect of climate is more pronounced for Home with a clear distinction between tropical and subtropical. For panel c), note that larger pumice stones are represented by larger circles and warmer colors, while smaller and cooler colored circles indicate smaller pumice clasts, and the effect of area is more distinct for the Home event (right‐hand cluster)

Community composition of pumice rafted biota that was nested within age, event, and climatic zone was found to have a significant interaction (Pseudo‐F = 3.8539, *p = *.00), while no distinct interaction between climatic zone and age or area was found (see Table S2). Community composition also differed depending on climatic zones (Pseudo‐F = 2.4565, *p = *.01) (see Table S2). Visualization of the influence of climatic zone using NMDS to compare the two events shows some differentiation particularly in regard to the Home event where tropical clasts were clearly differentiated from subtropical, this is not as apparent for Havre and this distinction may in part be due to the low number of collection points in the tropical climatic zone surveyed for the Home event (see Figure 3, panel b). The influence of climate on pumice raft community assembly is suspected to be related to the dispersion (beta diversity) of community composition. Tests of climatic zone dispersion were conducted for the Havre event (see Table S3) with greatest dispersion found between tropical and temperate climatic zones (*t *=* *3.4288, *p = *.01), followed by subtropical and temperate (*t *=* *2.5107, *p = *.02), while subtropical and tropical dispersion (*t *=* *1.4796, *p = *.19) were similar. Assessment of the dominance of epibiont groupings by climatic zone for both Home and Havre (see Table S6) indicates a change in species composition and associated dominance of species as climatic zone was altered.

### 3.3 Does pumice rafted community assembly (beta diversity) change for pumice that originated from a different eruption and that took a different trajectory?

**Figure 4:**
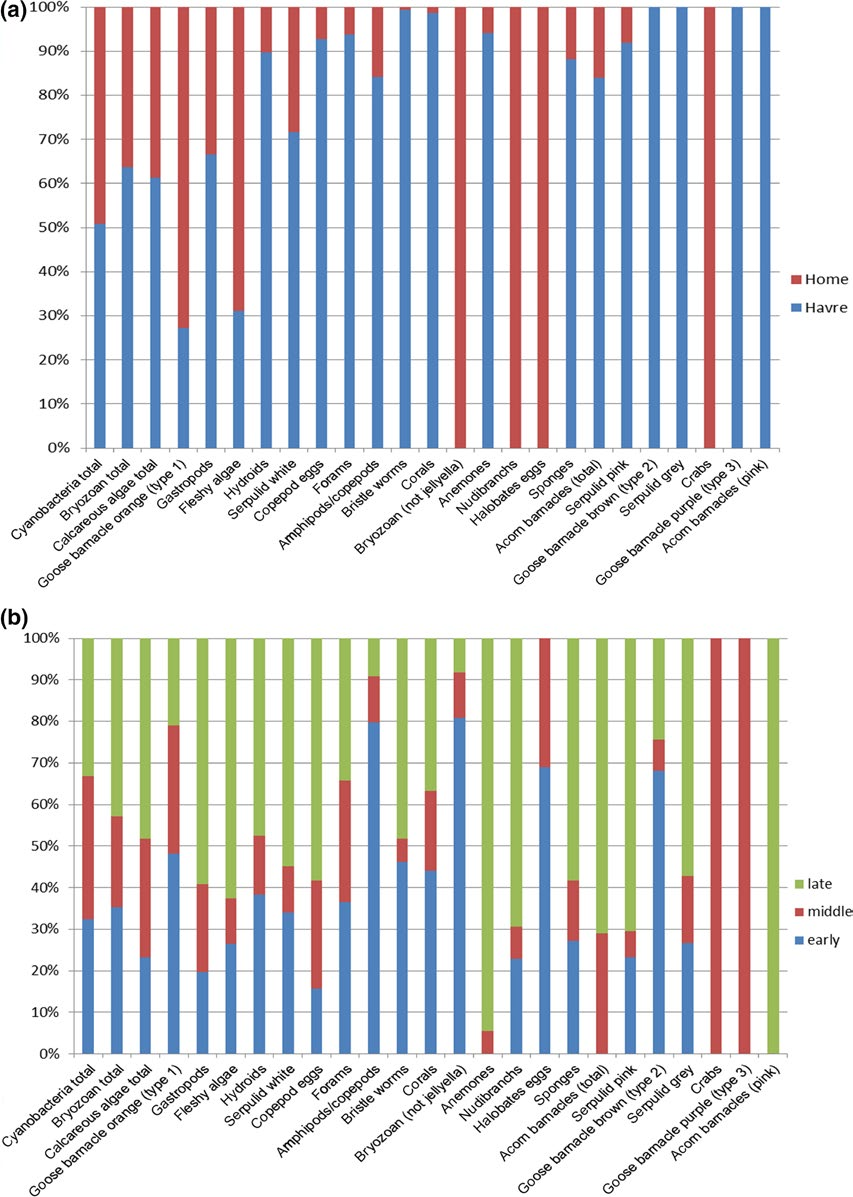
Percent dominance of different epibiont groupings a) by event (Home, *n* = 4,547*) (Bryan et al., 2012) versus (Havre, *n* = 403) and b) stage of pumice clast arrival for the combined data of Home and Havre): early (*n* = 265), middle (*n* = 3,944) and late (*n* = 741)
*Note: A reduced number of clasts was analyzed to produce these graphs for the Home event as species had to be aligned and combined between the two datasets. As there was a significant time lapse between collections and also different authors, some data were excluded due to the inability of ensuring the correct species were aligned in both datasets

We compared community change between pumice rafts from different origins with trajectories through differing climatic zones to determine whether differences in community composition or beta diversity occurred as a result. Overwhelmingly, the origin of the pumice raft (Home vs. Havre) is the most important main effect in our results (Pseudo‐F = 58.823, *p = *.00) (see Table S2 and Figure S2), followed closely by area (log_sphere) (Pseudo‐F = 28.82, *p = *.00); the interaction of area and event (log.sphere × event) (Pseudo‐F = 6.551, *p = *.00); the location of collection which is nested within age, event, and climatic zone (Pseudo‐F = 3.8539, *p = *.00); age (Pseudo‐F = 3.9378, *p = *.00); and climatic zone (Pseudo‐F = 2.4565, *p = *.01) (see Table S2). While overall a majority of pumice clast epibiont assemblages, regardless of climatic zone or event, were dominated by cyanobacteria, bryozoans, and calcareous algae, there are obvious differences in community structure and species between the two events of Home and Havre (see Table S7 and Figure 4, panel a). For example, the Home raft contained *Halobates* spp. eggs (a marine insect), nudibranchs, and crabs, where the Havre event did not. The Havre event meanwhile contained *Megabalanus coccopoma* (Darwin, 1854) an invasive acorn barnacle, gray serpulid worms, and two additional functional types of *Lepas* species that the Home event did not.

Examination of community composition indicated that pumice clast area was the second most important explanatory factor in our data (Pseudo‐F = 28.82, *p = *.00) (see Table S2). Visualization of this effect using an NMDS analyses shows most clearly the effect of pumice clast size for the Home event with larger clasts having different community composition; this same relationship between community composition and clast size is not as clear in the Havre event (see Figure 3, panel c).

Epibiont community composition was analyzed for the combined datasets of Home and Havre and was noted to change dependent upon the age of the pumice clasts (see Figure 4, panel b) with later arrivals being dominated by acorn barnacles. Middle arrivals were dominated by crabs and anemones, while early arrivals had a fairly even spread of community diversity indicating that through time diversity changed and increased. This is reflected in the pair‐wise tests conducted for the events separately, for the Havre event the largest difference in community composition was found between the early and late arrivals (*t *=* *2.1523, *p = *.00, see Table S4). For the Home event, this trend differed with the largest difference in community composition being between late and middle arrivals (*t *=* *2.1523, *p = *.00). Overall (both events combined) area had the strongest interactive effect with event (Pseudo‐F = 6.551, *p = *.00) closely followed by the location of pumice clast arrival, which was nested in age, event, and climatic zone (Pseudo‐F = 3.8539, *p = *.00) (see Table S2). Visualization of the effect of age (see Figure 3) shows that while age has an effect, this is also affected by the climatic zone encountered. For example, when examining the Home event, tropical clasts while being “young” in age had significantly different biota to other climatic zones.

## 4 DISCUSSION

In this study, we tested the SAR and elements of the GDM (i.e., age) on thousands of floating pseudo‐islands (i.e., floating pumice clasts) having the same substrate composition that were created on the same day and location for the respective volcanic eruptions (Bryan et al., 2012; Whittaker et al., 2008). Overall, we found evidence in support of the SAR and other abiotic drivers (i.e., age and climatic zone) that larger and older pumice clasts had higher epibiont richness (MacArthur & Wilson, 1963; Whittaker et al., 2008), while the influence of raft trajectory through different climatic zones caused changes in species assembly likely because of differences in the exposure of rafts to biota and climatic conditions such as water temperature (Thiel & Haye, 2006). While age and area were found to correlate positively with species richness, the influence of abiotic conditions such as temperature that limit many marine species distributions and increase the dominance of others should be considered when assessing the processes that explain distributions and richness of biota in isolated habitats (see, e.g., Wichmann et al., 2012).

### 4.1 Do area and age predict epibiont richness and does one have a stronger influence?

Older pumice clasts and those with a larger surface area were found to have higher epibiont richness when data from both Home and Havre volcanic eruptions were considered (MacArthur & Wilson, 1963; Whittaker et al., 2008). Larger habitat areas were also found to support increased species diversity in the plastic rafted communities of the Northern Pacific Ocean, attributing this to larger objects having greater stability in the water column in addition to simply increased habitat area (Goldstein et al., 2014).

### 4.2 How does the influence of area and age change for pumice clasts that were collected from different climatic zones?

We found that both subtropical and tropical rafts had strong positive relationships between epibiont richness and age and area, while epibiont richness on temperate rafts showed a positive relationship with area with age becoming negative. When we compared the community assembly forming on pumice clasts of different age, size, and climatic zone, we found that area had the strongest influence on final community assembly followed by age and climatic zone. Unsurprisingly, the effect of combining these factors showed larger, older clasts from the same event and clasts which remained in the same climatic zone for longer periods, collected at the same time from the same location had epibiont communities that differed the most from other samples. Other studies have found that time spent under certain abiotic conditions (e.g., rafting with changes of water temperature relating to season and latitude) affects the number and types of organisms available from the species pool to colonize and subsequently survive on the surface of floating or immersed objects (Anderson, 1999; Osman, 1978; Wichmann et al., 2012).

Species assembly of pumice clasts was most influenced by the pumice origin (Home vs. Havre) of the pumice clasts, followed by area and climatic zone. Pumice rafted communities (regardless of trajectory or age) were dominated by marine fouling communities including cyanobacteria, bryozoans, and calcareous algae which occurred fairly evenly regardless of time passing. Other biota increased in the middle to late time periods including anemones and acorn barnacles, while crabs and a functional type of goose barnacle occurred in the middle time period, and while *M. coccopoma* occurred only in the late stages and the climatic zone temperate. These differences may also be attributed to the origin of the pumice (Home vs. Havre) with the two different events having overwhelmingly different species present. Differences occurred between the three climatic zones such as the types of acorn barnacles observed increased for temperate clasts, while the presence of corals increased on tropical and subtropical clasts compared to temperate. Community composition or beta diversity differed between clasts from the Havre event with differing climatic zones collected, with the largest differences in community assembly being found between tropical and temperate, followed by subtropical and temperate. These differences were noted to be due to the dispersion of the diversity present on the clasts; for example, temperate clasts were significantly different from both tropical and subtropical communities in terms of their beta diversity, while we had no evidence for a difference between tropical and subtropical clasts (Anderson, 2006). This observation could be attributed to the different abilities of biota to survive in warmer or cooler water. Species richness of soft sediment dwelling invertebrates is known to decrease from the subtropics to the Arctic, but in the Southern Hemisphere where biodiversity hotspots frequently occur in cooler water as latitude increases, these same trends have been found not to hold at all spatial scales (Gray, 2002). Further to this, a study that mapped global distribution of species inhabiting coral reefs found a cline from the tropics to the poles for corals, molluscs, reef fish, and lobsters, providing evidence that a biological threshold for reef‐dwelling organisms comes into effect as water becomes cooler (Roberts et al., 2002). At the same time, we acknowledge that the reduced number of clasts collected for both tropical and temperate climatic zones may have caused some of the observed results.

### 4.3 Does pumice rafted community assembly change for pumice that originated from a different eruption and that took a different trajectory?

Our results also suggest that raft trajectory into different climatic zones may cause a die‐off and change in community assembly. Our finding that species are lost through time as pumice enters cooler waters indicates that water temperature change as a result of either season or latitude can cause a change in the numbers and types of epibionts able to colonize and survive on pumice rafted at different times of the year (a seasonality effect) and to different latitudes (a climatic zone effect) which are both influenced by the underlying local species pool (Anderson et al., 2006; Mayfield & Levine, 2010; Thiel & Haye, 2006; Wichmann et al., 2012). This result is supported in similar studies whereby recruitment onto submerged habitats of different sizes in one location was affected by the time of year (season) and the size of the available habitat space (see Anderson, 1999; Anderson & Underwood, 1994; Osman, 1978). Temperature can directly influence the reproduction of certain marine epibionts as recruitment by different species has been observed to occur at different times of the year (see Anderson, 1999; Anderson & Underwood, 1994; Osman, 1978), while habitat size affected the stability and niche space of the experimental habitats with larger habitats recruiting higher species diversity (Osman, 1978). Even relatively small changes in latitude, for example, from north to south of a coastline have been shown to cause shifts in community composition (due to shifts from warmer to cooler waters) (see Wichmann et al., 2012) or restrictions in the presence of rafting biota at different latitudes regardless of the presence of suitable rafting substrate (see Thiel & Haye, 2006; Thiel et al., 2003). In addition to the effect of temperature, it is also possible that clasts collected from the temperate climatic zone had fewer shallow reef or island encounters on their voyage, and hence, species were lost due to reduced levels of recolonization from source populations (Brown & Kodric‐Brown, 1977). For example, Brown and Kodric‐Brown (1977) found that extinctions of invertebrates on thistle plants were related to distances to source populations (i.e., isolation), and hence, recolonization of species already present on plants from proximal sources saved some populations from potential extinctions resulting in what they term the “rescue effect.”

We also found Havre pumice that came ashore in temperate water, had subtropical species recruitment (e.g., corals), which while being present may have ceased growth and died as rafts dispersed into cooler water, while other species, for example, acorn barnacles increased in dominance and overgrew other epibionts under these conditions. In addition, several species found on the Havre pumice have only been recorded as present in warmer waters of tropical and subtropical climes but for the first time were documented washing up in the temperate water of Tasmania these included two species of pearl‐oyster (*Pinctada margaritifera* and *Pinctada sugillata*) and the gastropod (*Litiopa limnophysa*) (as found by Grove, 2014) and the corals *Porites lobata* and an *Acropora* spp. as found in examination of Havre pumice from Tasmania examined in this study (Veron & Stafford‐Smith, 2000). Although these animals were dead upon collection and it would be difficult to know whether they died due to desiccation upon pumice stranding on the beach or due to the cooler temperature of the waters encountered, this information supports the trend that we have observed in the data of a change in assembly as pumice drifted further south and a die‐off in species resulting in a negative relationship between age and epibiont richness. Also found in Havre temperate pumice was the invasive species of acorn barnacle *M. coccopoma,* a native of the tropical eastern Pacific (Yamaguchi et al., 2009). This species is already recorded as present in Australia and possibly arrived initially via shipping, although in Tasmania records indicate presence on the hull of a ship and not an established population (Yamaguchi et al., 2009). The presence of *M. coccopoma* highlights the importance of studying and further understanding the phenomenon of pumice rafting. Many introductions of exotic species have been attributed to shipping as the main source of recruits, and however, other rafting substrates such as pumice and plastic are proving to be significant contributors to marine biota transportation due to their volume and persistence in the water column (Bryan et al., 2012; Goldstein et al., 2014). *M. coccopoma* has already spread to Brazil, Europe, Japan, and California, providing evidence of its ability to colonize, reproduce, and spread in new habitats after transportation is a significant concern for managers of shallow marine ecosystems in Australia (Yamaguchi et al., 2009).

We cannot be certain as to whether sessile pumice rafted biota such as corals, bryozoans, and barnacles from the Home and Havre rafts would then colonize habitats when they arrive on coastlines; however, studies have shown that colonization is possible (Jokiel, 1989, 1992). Three possible mechanisms of coral rafted on pumice to colonize a new habitat are proposed (after Jokiel, 1989): (1) the chance sinking of overladen pumice into shallow water allowing fouling organisms to overgrow (see Tunnicliffe, 1981 for evidence of loose coral fragments being overgrown and secured by calciferous reef organisms); (2) dislodgement via scraping of pumice fragments on tidal reef flats via wave action (see Smith & Hughes, 1999; Tunnicliffe, 1981); and (3) mature colonies reproducing at sea either asexually (via budding, parthenogenesis, or polyp bailout) or sexually by eggs that are fertilized either before or after being released by the parent (see Hoeksema, Roos, & Cadee, 2011; Sammarco, 1982; Stoddart, 1983), which can be possible even if only one coral colony is present as many corals are self‐fertile (Combosch & Vollmer, 2013; Hoeksema et al., 2011; Jokiel, 1989; Sammarco, 1982; Stoddart, 1983). While the temperate waters of Tasmania are too cold presently for corals, it is possible that as sea temperatures rise the expansion of coral species into new climatic zones could occur. The spread and contraction of coral species into new habitats both now and historically has recently been documented in several studies (see Fenner & Banks, 2004; Greenstein & Pandolfi, 2008; Hoeksema et al., 2011; Mantelatto, Creed, Mourão, Migotto, & Lindner, 2011; Precht & Aronson, 2004; Yamano, Sugihara, & Nomura, 2011), which suggests that coral rafted on pumice may have an opportunity to escape unfavorable conditions (e.g., hotter water) via rafting as global temperatures rise (Greenstein & Pandolfi, 2008; Hoeksema et al., 2011).

The results of this study were found despite an inability to identify all of the biota inhabiting the pumice clasts, and the departure of motile biota (such as crabs, bristle worms, gastropods, and nudibranchs), which disembarked pumice clasts upon stranding and were often not captured during collection of pumice samples. The departure of motile biota will have reduced the effect of area on species richness, and as a result, we think our finding of a positive relationship between area and epibiont richness would have been made even stronger should we have been able to capture this biota in our data as part of the study. In addition, we acknowledge that there is no way of knowing if clasts from the “older” collections in the temperate zone were less diverse in species assemblage due to potential sinking of more diverse assemblages; however, as older clasts from tropical and subtropical were quite diverse, we assume we captured range of diversity from each climatic zone and stage of arrival. Despite these limitations, we were able to demonstrate the importance of pumice rafting as a mass dispersal agent of marine biodiversity throughout the Pacific Ocean and beyond (Bryan et al., 2012; Nikula, Fraser, Spencer, & Waters, 2010). For example, many species (e.g., corals) found on the surface of pumice clasts disperse by zooplankton and spawning and have documented maximum dispersal limits ranging from a few meters to several hundred kilometers and yet the same species of coral are found in reefs separated by thousands of kilometers throughout the Pacific ocean (Jokiel, 1989; Shanks, Grantham, & Carr, 2003; Thiel & Haye, 2006). A similar observation was made (see Nikula et al., 2010) whereby a genetic study of nondispersive crustaceans which are known kelp rafters were sampled throughout the islands of the subantarctic. These crustaceans raft on kelp throughout the circumpolar currents of the subantarctic and were found to come from the same haplotype providing evidence that kelp rafting contributes substantially to the composition of shallow marine communities in the subantarctic despite being separated by hundreds to thousands of kilometers (Nikula et al., 2010).

Overall pumice raft events have provided strong evidence in support of the key tenets within TIB, namely the SAR and the inclusion of additional abiotic and biotic drivers in models of species richness for example the GDM which predicts that age and area will be the major driving force in predicting biodiversity in insular habitats (MacArthur & Wilson, 1963, 1967; Whittaker et al., 2008). This study contains a line of evidence that these theories have continued relevance to the understanding of ecological communities and the abiotic and biotic processes which shape them (Keppel et al., 2010; MacArthur & Wilson, 1967; Whittaker et al., 2008).

## CONFLICT OF INTEREST

None declared.

## AUTHOR CONTRIBUTIONS

EV, JF, and SEB designed the study. EV collected the data for the Havre eruption, developed the models, performed the analysis, and wrote the first draft of the manuscript. JF assisted with both statistical analysis and manuscript development. SEB collected the data for the Home Reef eruption. ME, AGC, and LH all assisted with marine biota identification. SEB, ME, AGC, and LH all contributed to editing the manuscript.

## DATA ACCESSIBILITY STATEMENT

Data available from the Dryad Digital Repository: https://doi.org/10.5061/dryad.20r509g .
