# Supplementary material for: Age and area predict patterns of species richness in pumice rafts contingent on oceanic climatic zone encountered
Source: Ecol Evol. 2018 Apr 24;8(10):5034–46. doi: 10.1002/ece3.3980 (PMC5980578; doi:10.1002/ece3.3980)
Supplement: Supplementary file 1 [file ECE3-8-5034-s001.docx]

**Supporting Information**

**Appendix S1. Pumice characteristics expanded**

Pumice is essentially a magma foam that is rapidly chilled on eruption and ejection into the atmosphere or water which quenches the magma to glass. The high proportion of vesicle or bubble spaces in the glass reduces its bulk density so that it is less than water, and allows the pumice stone to float in water. Many of the vesicle spaces in each pumice clast remain unconnected preventing occupation of those spaces by water aiding long-term positive buoyancy. Consequently, the combination of long-term positive buoyancy and resistance to physical/chemical degradation or biological consumption provides countless opportunities for marine epibionts to colonise pumice and undergo mass transit across deep oceans ([Thiel & Haye 2006](#_ENREF_7); [Bravo *et al.* 2011](#_ENREF_1)).

At a macroscopic scale, pumice appears relatively homogenous. However, significant habitat heterogeneity exists in each clast. First, the foam like texture of pumice greatly increases the surface area for attachment, as well as depressions and holes that offer value as protective spaces such as for newly settled larvae ([Bryan *et al.* 2004](#_ENREF_2); [Bravo *et al.* 2011](#_ENREF_1)). Second, based on the way pumice clasts float in water, many clasts are able to maintain stability in the water column with some exhibiting emergent dorsal surface or freeboard directly exposed to sunlight, and a ventral surface fully submerged and shaded ([Bryan *et al.* 2012](#_ENREF_3)). Importantly, these two habitat types allow species with different requirements to populate each area, photosynthetic organisms can flourish on dorsal surfaces, and filter feeders on ventral surfaces, thereby promoting greater biodiversity on each clast ([Bryan *et al.* 2012](#_ENREF_3)). Differences in buoyancy (whether maintaining dorsal surface above or below the water line), available habitat space and floating stability between smaller and larger clasts may also influence patterns of species richness that assemble on the pumice casts ([MacArthur & Wilson 1967](#_ENREF_5); [Osman 1978](#_ENREF_6); [Bravo *et al.* 2011](#_ENREF_1); [Goldstein *et al.* 2014](#_ENREF_4)).

Pumice producing eruptions can occur worldwide but occur most commonly in the South-western Pacific, with floating pumice arriving every five to ten years on the eastern Australian coastline for at least the last 200 years ([Bryan 1971](#_ENREF_13); [Bryan *et al.* 2004](#_ENREF_11); [Bryan *et al.* 2012](#_ENREF_12)). Each pumice clast is a self-contained fragment of pumice stone with variable size and shape. As pumice clasts float through the ocean, they are acted on by abiotic forces including temperature, wind and waves, causing altered trajectories and exposure to changes in temperature and colonising propagules. For example, the raft may remain in the deep ocean isolating it from potential colonising propagules or have close encounters with islands, reefs and other shallow marine ecosystems causing it to be bombarded by colonising populations ([Jokiel 1989](#_ENREF_37); [Bravo *et al.* 2011](#_ENREF_10); [Bryan *et al.* 2012](#_ENREF_12)). Pumice stones may float (until inevitable stranding on shorelines or sinking due to biofouling or waterlogging) for more than two years and can travel more than 20,000 kilometres ([Jokiel 1989](#_ENREF_37); [Risso *et al.* 2002](#_ENREF_56); [Bravo *et al.* 2011](#_ENREF_10); [Bryan *et al.* 2012](#_ENREF_12)).

**Table S1. Pumice stranding collection field sites for both the Home and Havre events - including latitude, longitude and date of collection.**

| **Volcano** | **Sample site** | **Latitude** | **Longitude** | **Sampling date** |
| --- | --- | --- | --- | --- |
| Home | Vava’u islands Tonga | 18°S 39'40.34" | 174°W 3'15.79" | 01/02/2007 |
| Home | Marion Reef | 19˚S 05.744’ | 152˚E 23.449’ | 30/04/2007 |
| Home | Lamberts Beach | 21˚S 04.472’ | 149˚E 3.701’ | 01/05/2007 |
| Home | Lady Musgrave Island | 23˚S 54.461’ | 152˚E 3.669’ | 03/05/2007 |
| Home | Agnes Waters | 24˚S 12.463’ | 151˚E 54.364’ | 03/05/2007 |
| Home | South Stradbroke Island | 27˚S 49.678’ | 153˚E 5.968’ | 01/06/2007 |
| Home | Broadbeach, Gold Coast, Queensland | 28˚S 07.620’ | 153˚E 26.135’ | 05/05/2007; 27/12/2007; 21/01/2008 |
| Home | Duranbah | 28˚S 10.005’ | 153˚E 33.105’ | 05/05/2007 |
| Home | Byron Bay, Queensland | 28˚S 38.334’ | 153˚E 37.636’ | 05/05/2007 |
| Home | Shelley Beach, Ballina | 28˚S 51.598’ | 153˚E 5.795’ | 05/05/2007 |
| Havre | Bicheno, Waubs Beach, Tasmania | 41°S 52'20.11" | 148°E 17'57.08" | 08/04/2014; 30/04/2014 |
| Havre | Schouten Island, Tasmania | 42°S 19'3.91" | 148°E 18'55.74" | 07/04/2014 |
| Havre | Scamander River Mouth, Tasmania | 41° S 27'41.55" | 148°E 15'56.84" | 07/04/2014; 06/06/2014 |
| Havre | Flinders Island, Trousers Point Beach, Tasmania | 40°S 13'47.24" | 148°E 2'23.93" | 10/06/2014 |
| Havre | Flinders Island, Emita Foreshore, Tasmania | 39°S 59'55.88" | 147°E 53'48.06" | 21/06/2014 |
| Havre | Safety Cove, Port Arthur, Tasmania | 43°S 9'45.85" | 147°E 51'18.06" | 27/06/2014 |
| Havre | Vomo Island, Fiji | 17°S 30'17.01" | 177°E 16'1.30" | 02/09/2013; 05/09/2013 |
| Havre | Main Beach, Port Douglas, Queensland | 16°S 29'24.15" | 145°E 27'54.06" | 24/08/2013 |
| Havre | Lowe Isle, The Lowe Isles, Queensland | 16°S 23'2.66" | 145°E 33'36.60" | 25/08/2013 |
| Havre | Shoal Point Beach, Mackay, Queensland | 21°S 0'50.77" | 149°E 9'26.45" | 01/09/2013 |
| Havre | Main Beach, Noosa, Queensland | 26°S 23’15.97” | 153°E 5’16.33” | 23/12/2013 |
| Havre | Prince of Wales Island, Torres Strait | 10°S 42’49.40” | 142°E 11’54.18” | 16/09/2013 |
| Havre | Grassy Head Beach, NSW | 30°S 46’49.86” | 152°E 59’48.97” | 01/01/2014 |
| Havre | Gold Coast, Burleigh, Queensland | 28°S 5’19.15” | 153°E 27’12.78” | 30/03/2013; 28/12/2013 |
| Havre | The Spit, Gold Coast, Queensland | 27°S 56'21.53" | 153°E 25'45.77" | 12/12/2013 |
| Havre | North Stradbroke Island, Queensland | 27°S 25’28.97” | 153°E 32’17.20” | 01/05/2013 |
| Havre | Deadman’s Beach, North Stradbroke Island, Queensland | 27°S 25’30.60” | 153°E 32’21.62” | 01/05/2013; 05/05/2013 |
| Havre | Frenchman’s Beach, North Stradbroke Island, Queensland | 27°S 25’38.85” | 153°E 32’35.23” | 04/05/2013; 02/07/2013; 04/08/2013 |
| Havre | Main Beach, North Stradbroke Island, Queensland | 27°S 26'13.72" | 153°E 32'32.17" | 02/07/2013; 04/08/2013 |
|  |  |  |  |  |

**Table S2. PERMANOVA test of differences between pumice rafted communities which formed on pumice from different events (Home versus Havre), locations, ages, sizes and climatic zones (trajectories): subtropical (n=4719), tropical (n=161) and temperate (n=70).**

| **comparison of event, location, age, area and climatic zone** | **Pseudo-F** | ***P*** | **Unique permutations** |
| --- | --- | --- | --- |
| event | 58.823 | 0.0001 | 9958 |
| area (log.sphere) | 28.82 | 0.0001 | 9939 |
| area (log.sphere) x event | 6.551 | 0.0001 | 9940 |
| age | 3.9378 | 0.0005 | 9931 |
| location (age x event x climaticzone) | 3.8539 | 0.0001 | 9826 |
| climate | 2.4565 | 0.0062 | 9935 |

**Table S3. PERMDISP analysis for the Havre event and climatic zone providing a distance-based test for homogeneity of multivariate dispersions between climatic zones of tropical (n=116), subtropical (n=218) and temperate (n=70) (NB: the number of permutations was set to 9999 for all models).**

| **climatic zone** | **t value** | ***P*** |
| --- | --- | --- |
| tropical, temperate | 3.4288 | 0.0064 |
| subtropical, temperate | 2.5107 | 0.0243 |
| subtropical, tropical | 1.4796 | 0.187 |

**Table S4. Pair-wise comparison tests of pumice rafted epibiont communities by age and separated by event.**

| **Havre** |  |  |  |
| --- | --- | --- | --- |
| **time of arrival (age) compared** | **t value** | ***P*** | **Unique permutations** |
| late, early | 2.1523 | 0.0001 | 9945 |
| late, middle | 0.98867 | 0.4791 | 9953 |
| middle, early | 0.84993 | 0.6369 | 9938 |
| **Home** |  |  |  |
| late, middle | 2.7911 | 0.0001 | 9789 |
| late, early | 1.2514 | 0.4154 | 120 |
| middle, early | 1.0534 | 0.3067 | 9721 |

**Table S5. Counts and presence data for epibiont groupings divided into respective oceanic climatic zones, combined for the two events of Home and Havre.**

|  | **Oceanic climatic zone** | | |  |
| --- | --- | --- | --- | --- |
|  | **temperate** | **subtropical** | **tropical** | **Grand Total** |
| **Epibiont counts of individuals per clast** | | | | |
| *Lepas* spp. #1 | 0 | 1098 | 21 | 1119 |
| *Lepas* spp. #2 | 0 | 6 | 5 | 11 |
| *Lepas* spp. #3 | 0 | 0 | 4 | 4 |
| *Lepas* spp. juveniles (<0.5cm) | 47 | 147 | 85 | 279 |
| Acorn barnacles | 11 | 18 | 7 | 36 |
| *Megabalanus coccopoma* | 6 | 0 | 0 | 6 |
| Juvenile acorn barnacles | 0 | 13 | 0 | 13 |
| Sea anemones | 1 | 30 | 3 | 34 |
| Chitons | 0 | 0 | 1 | 1 |
| Copepod eggs (denoting presence of copepods) | 29 | 147 | 39 | 215 |
| Crabs | 0 | 4 | 0 | 4 |
| Shrimp | 0 | 4 | 0 | 4 |
| Bristle worms | 14 | 45 | 10 | 69 |
| Serpulidae type. #1 | 9 | 251 | 41 | 301 |
| Serpulidae type. #2 | 8 | 12 | 7 | 27 |
| Serpulidae type. #3 | 4 | 4 | 8 | 16 |
| Nudibranchs | 0 | 29 | 0 | 29 |
| Amphipods and Copepods | 2 | 57 | 16 | 75 |
| Ostracods | 0 | 13 | 0 | 13 |
| *Halobates* spp. #1 | 0 | 23 | 0 | 23 |
| Foram type #1 | 0 | 1 | 4 | 5 |
| Foram type #2 | 0 | 2 | 47 | 49 |
| Foram type #3 | 0 | 1 | 33 | 34 |
| Foram type #4 | 0 | 3 | 18 | 21 |
| Foram type #5 | 15 | 27 | 26 | 68 |
| Foram type #6 | 0 | 1 | 11 | 12 |
| Foram type #7 | 0 | 20 | 4 | 24 |
| Foram type #8 | 2 | 68 | 0 | 70 |
| *Pteria* spp. #1 | 1 | 4 | 3 | 8 |
| *Electroma* spp. #1 | 0 | 11 | 0 | 11 |
| *Pinctada margaritifera* | 0 | 15 | 9 | 24 |
| *Litiopa limnophysa* | 8 | 673 | 28 | 709 |
| *Epitoniid* spp. #1 | 0 | 15 | 0 | 15 |
| *Rissoidean* spp. #1 | 0 | 4 | 0 | 4 |
| *Rissoidean* spp. #2 | 0 | 0 | 1 | 1 |
| *Dalia* spp. #1 | 0 | 1 | 0 | 1 |
| *Janthia* spp. #1 | 0 | 1 | 0 | 1 |
| *Janthia* spp. #2 | 0 | 1 | 1 | 2 |
| *Nerita* spp. #1 | 0 | 1 | 0 | 1 |
| *Crassostrea* spp. #1 | 0 | 6 | 1 | 7 |
| *Crassostrea* spp. #2 | 0 | 6 | 0 | 6 |
| *Crassostrea* spp. #3 | 0 | 1 | 0 | 1 |
| Gastropod type #1 | 0 | 0 | 9 | 9 |
| Gastropod type #2 | 0 | 0 | 3 | 3 |
| Gastropod type #3 | 0 | 1 | 9 | 10 |
| Gastropod type #4 | 0 | 2 | 9 | 11 |
| Gastropod type #5 | 0 | 0 | 1 | 1 |
| Gastropod type #6 | 0 | 1 | 0 | 1 |
| Gastropod type #7 | 0 | 0 | 5 | 5 |
| Gastropod type #8 | 3 | 0 | 0 | 3 |
| *Hiatella australis* | 0 | 4 | 4 | 8 |
| Juvenile *Pinctada* spp. #1 | 0 | 1 | 6 | 7 |
| Juvenile *Pinctada* spp. #2 | 0 | 2 | 0 | 2 |
| *Brachidontes subramosa* | 0 | 2 | 1 | 3 |
| *Anomiid* spp. #1 | 0 | 3 | 0 | 3 |
| *Septifer australis* | 0 | 2 | 0 | 2 |
| Bivavlia type #1 | 0 | 1 | 1 | 2 |
| Bivalvia type #2 | 0 | 0 | 1 | 1 |
| *Pinctada fucata* | 0 | 0 | 1 | 1 |
| Sponges | 0 | 14 | 1 | 15 |
| *Pocillopora* spp. #1 | 0 | 24 | 22 | 46 |
| *Acropora* spp. #1 | 1 | 0 | 1 | 2 |
| *Porites lobata* | 1 | 3 | 0 | 4 |
| Juvenile corals (<0.1mm) | 0 | 6 | 1 | 7 |
| Brown crustacean (no ID possible)* | 1 | 0 | 0 | 1 |
| Pink marine mite (no ID possible)* | 0 | 0 | 1 | 1 |
| Grey lifeform (whip-like) (no ID possible)* | 1 | 2 | 0 | 3 |
| Clear lifeform, brown margins (no ID possible)* | 0 | 0 | 1 | 1 |
|  |  |  |  |  |
|  |  |  |  |  |
| **Colonial epibionts - presence data per clast** | | | | |
|  | | | | |
| Bryozoan - *Jellyella* spp. | 65 | 2107 | 92 | 2264 |
| Bryozoan - type #1 | 0 | 29 | 0 | 29 |
| Bryozoan - type #2 | 0 | 0 | 13 | 13 |
| Bryozoan - type #3 | 0 | 0 | 2 | 2 |
| Bryozoan - type #4 | 0 | 0 | 1 | 1 |
| Bryozoan - type #5 | 0 | 0 | 2 | 2 |
| Bryozoan - type #6 | 0 | 3 | 0 | 3 |
| Cyanobacteria - Nostocales spp. #1 | 70 | 269 | 116 | 455 |
| Cyanobacteria - Rivularia spp. #1 | 61 | 849 | 65 | 975 |
| Cyanobacteria - Rivularia spp. #2 | 0 | 4089 | 22 | 4111 |
| Cyanobacteria spp. #4 | 0 | 22 | 0 | 22 |
| Cyanobacteria - Oscillatoriales spp. #1 | 0 | 714 | 1 | 715 |
| *Caulerpa* spp. #1 | 3 | 63 | 0 | 66 |
| *Caulerpa peltata* | 0 | 3 | 0 | 3 |
| *Caulerpa razemosa* | 0 | 2 | 0 | 2 |
| *Caulerpa nammularia* | 0 | 5 | 0 | 5 |
| *Sargassum* spp. #2 | 0 | 0 | 9 | 9 |
| *Sargassum flavicans* | 0 | 36 | 0 | 36 |
| *Symploca* spp. #1 | 0 | 6 | 0 | 6 |
| *Ceramium* spp. #1 | 0 | 199 | 3 | 202 |
| *Ceramium* spp. #2 | 0 | 2 | 2 | 4 |
| Green algae spp. | 1 | 3 | 0 | 4 |
| Red algae spp. | 1 | 0 | 0 | 1 |
| *Cladophora* spp. #1 | 0 | 177 | 0 | 177 |
| *Chondria* spp. #1 | 0 | 1 | 0 | 1 |
| *Corrallina* spp. #1 | 0 | 1 | 0 | 1 |
| *Jania* spp. #1 | 0 | 14 | 1 | 15 |
| *Polysiphonia* spp. #1 | 0 | 28 | 1 | 29 |
| *Hypoglossum* spp. #1 | 0 | 2 | 3 | 5 |
| *Callithamnion* spp. #1 | 0 | 14 | 0 | 14 |
| *Colpomenia* spp. #1 | 0 | 1 | 0 | 1 |
| *Enteromorpha* spp. #1 | 0 | 0 | 1 | 1 |
| Hydrozoa type #1 | 33 | 140 | 46 | 219 |
| Hydrozoa type #2 | 7 | 225 | 21 | 253 |
| Hydrozoa type #3 | 4 | 3 | 7 | 14 |
| Scyphozoa spp. #1 | 0 | 3 | 4 | 7 |
| Calcareous algae type #1 | 28 | 40 | 40 | 108 |
| Calcareous algae type #2 | 30 | 51 | 59 | 140 |
| Calcareous algae type #3 | 1 | 1 | 8 | 10 |
| Calcareous algae type #4 | 10 | 50 | 17 | 77 |
| Calcareous algae type #5 | 2 | 344 | 43 | 389 |
| Calcareous algae type #6 | 1 | 10 | 0 | 11 |
| Calcareous algae type #7 | 0 | 1 | 0 | 1 |
| Calcareous algae type #8 | 1 | 0 | 0 | 1 |
| Calcareous algae type #9 | 0 | 1434 | 1 | 1435 |
| Calcareous algae type #10 | 0 | 22 | 0 | 22 |

*****Despite best efforts these four epibionts were not possible to identify although they had characteristics evident of marine species and also being distinct from other biota, as such they were included in the statistical modelling completed for this paper.

**Table S6. Percent dominance of major epibiont groupings for the combined events of Home and Havre, per pumice clast for the three climatic zones of subtropical (n=4719), tropical (n=161) and temperate (n=70).**

| **Epibiont grouping** | **subtropical** | **tropical** | **temperate** |
| --- | --- | --- | --- |
| cyanobacteria total | 96.9% | 85.7% | 100.0% |
| bryozoan total | 45.2% | 64.6% | 92.9% |
| calcareous algae total | 37.7% | 76.4% | 67.1% |
| goose barnacle orange | 23.3% | 13.0% | 0.0% |
| gastropods | 15.4% | 33.5% | 15.7% |
| fleshy algae | 10.0% | 9.9% | 4.3% |
| hydroids | 7.4% | 37.9% | 55.7% |
| serpulid white | 5.3% | 25.5% | 12.9% |
| copepod eggs | 3.1% | 24.2% | 41.4% |
| forams | 2.5% | 41.0% | 24.3% |
| amphipods/copepods | 1.2% | 9.9% | 2.9% |
| bristle worms | 1.0% | 6.2% | 20.0% |
| corals | 0.7% | 14.9% | 2.9% |
| bryozoan (excluding *jellyella*) | 0.7% | 8.7% | 0.0% |
| anemones | 0.6% | 1.9% | 1.4% |
| nudibranchs | 0.6% | 0.0% | 0.0% |
| halobates eggs | 0.5% | 0.0% | 0.0% |
| sponges | 0.3% | 0.6% | 0.0% |
| acorn barnacles (total) | 0.3% | 0.0% | 8.6% |
| serpulid pink | 0.3% | 4.3% | 11.4% |
| goose barnacle brown | 0.1% | 3.1% | 0.0% |
| serpulid grey | 0.1% | 5.0% | 5.7% |
| crabs | 0.1% | 0.0% | 0.0% |
| goose barnacle purple | 0.0% | 2.5% | 0.0% |
| acorn barnacles (pink) | 0.0% | 0.0% | 8.6% |

**Table S7. Epibiont grouping showing % dominance by pumice rafting event of Havre (n=403) and Home (n=4547). Note both events have large dominant groupings of cyanobacteria, bryozoans and calcareous algae.**

| **Epibiont grouping** | **Havre** | **Home** |
| --- | --- | --- |
| cyanobacteria total | 100.0% | 96.3% |
| bryozoan total | 76.7% | 43.8% |
| calcareous algae total | 59.6% | 37.6% |
| goose barnacle orange (type 1) | 8.9% | 23.8% |
| gastropods | 29.5% | 14.8% |
| fleshy algae | 4.7% | 10.4% |
| hydroids | 48.6% | 5.5% |
| serpulid white | 13.6% | 5.4% |
| copepod eggs | 28.3% | 2.2% |
| forams | 28.5% | 1.9% |
| amphipods/copepods | 6.0% | 1.1% |
| bristle worms | 15.6% | 0.1% |
| corals | 7.7% | 0.1% |
| bryozoan (not jellyella) | 0.0% | 1.0% |
| anemones | 5.0% | 0.3% |
| nudibranchs | 0.0% | 0.6% |
| halobates eggs | 0.0% | 0.5% |
| sponges | 1.5% | 0.2% |
| acorn barnacles (total) | 1.5% | 0.3% |
| serpulid pink | 6.7% | 0.6% |
| goose barnacle brown (type 2) | 2.7% | 0.0% |
| serpulid grey | 4.0% | 0.0% |
| crabs | 0.0% | 0.1% |
| goose barnacle purple (type 3) | 1.0% | 0.0% |
| acorn barnacles (pink) | 1.5% | 0.0% |

**
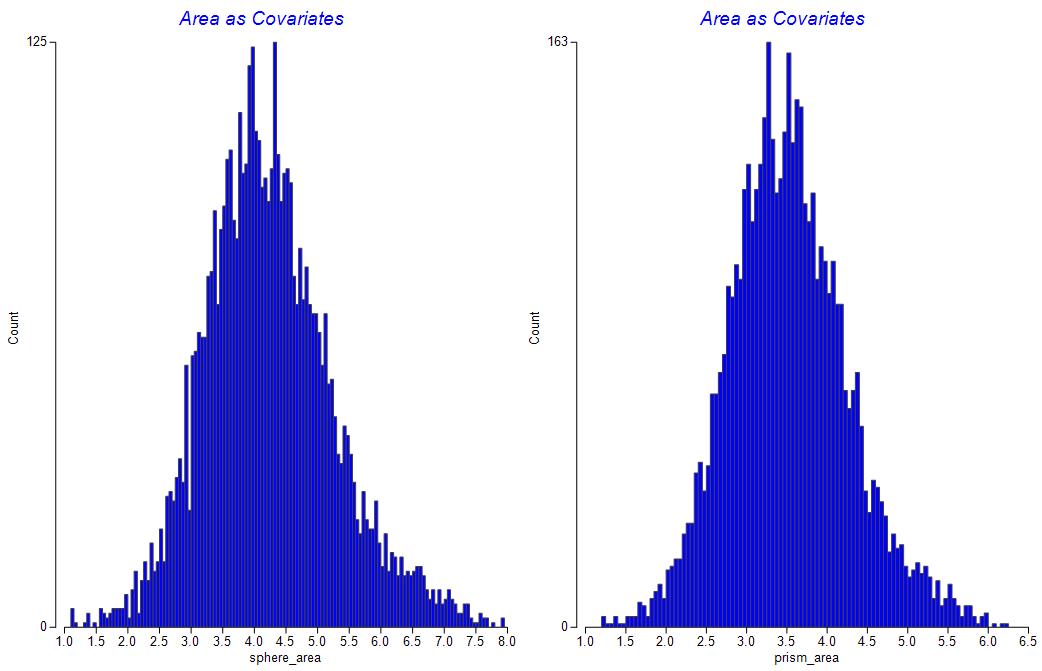
**

**Figure S2. Histogram of pumice clast sizes calculated as both a sphere and a prism.**

**
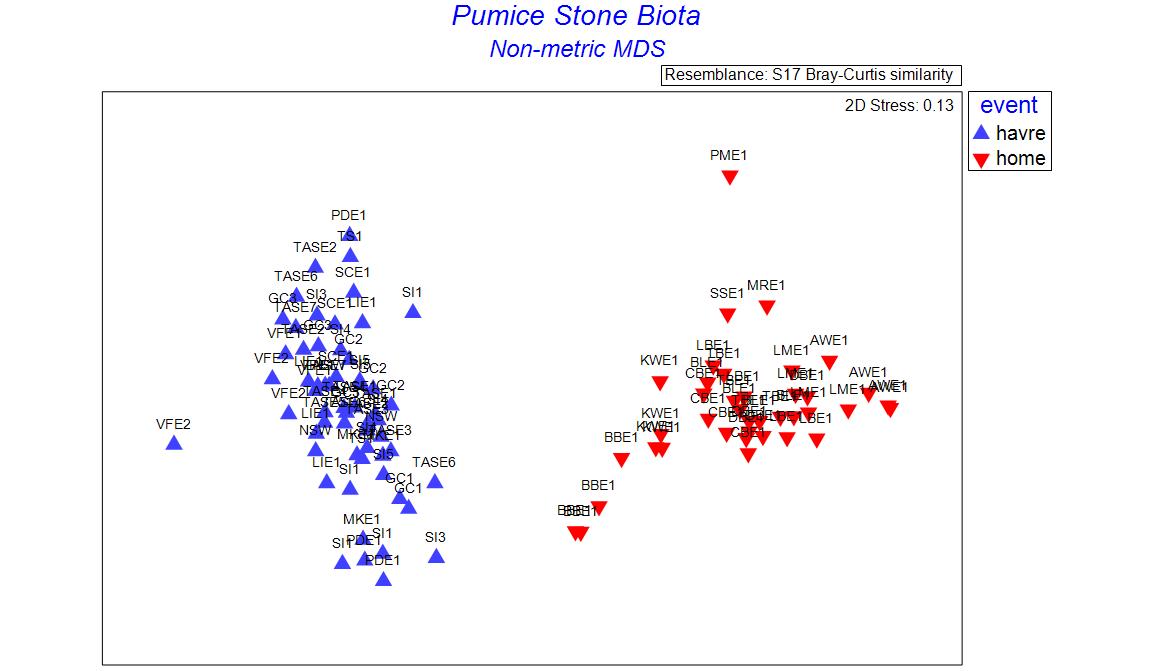
**

**Figure S1. NMDS of pumice rafted community composition by location for two events Home (red-coloured right-hand cluster) and Havre (blue-coloured left-hand cluster).**

**References**

Bravo M., Carlos Astudillo J., Lancellotti D., Luna-Jorquera G., Valdivia N. & Thiel M. (2011). Rafting on abiotic substrata: properties of floating items and their influence on community succession. *Marine Ecology Progress Series*, 439, 1.

Bryan S.E., Cook A., Evans J.P., Colls P.W., Wells M.G., Lawrence M.G., Jell J.S., Greig A. & Leslie R. (2004). Pumice rafting and faunal dispersion during 2001–2002 in the Southwest Pacific: record of a dacitic submarine explosive eruption from Tonga. *Earth and Planetary Science Letters*, 227, 135-154.

Bryan S.E., Cook A.G., Evans J.P., Hebden K., Hurrey L., Colls P., Jell J.S., Weatherley D. & Firn J. (2012). Rapid, Long-Distance Dispersal by Pumice Rafting. *PLoS One*, 7, e40583.

Goldstein M., Carson H. & Eriksen M. (2014). Relationship of diversity and habitat area in North Pacific plastic-associated rafting communities. *Mar Biol*, 161, 1441-1453.

MacArthur R.H. & Wilson E.O. (1967). *The Theory of Island Biogeography*. Princeton University Press, Princeton, New Jersey.

Osman R.W. (1978). The influence of seasonality and stability on the species equilibrium. *Ecology*, 59, 383-399.

Thiel M. & Haye P.A. (2006). The Ecology of Rafting in the Marine Environment. III. Biogeographical and Evolutionary Consequences. *Oceanography and Marine Biology: An Annual Review*, 44, 323-429.
